# Predicting capillary vessel network hemodynamics in silico by machine learning

**DOI:** 10.1093/pnasnexus/pgae043

**Published:** 2024-01-31

**Authors:** Saman Ebrahimi, Prosenjit Bagchi

**Affiliations:** Mechanical and Aerospace Engineering Department, Rutgers, The State University of New Jersey, Piscataway, NJ 08854, USA; Mechanical and Aerospace Engineering Department, Rutgers, The State University of New Jersey, Piscataway, NJ 08854, USA

**Keywords:** microcirculation, hemodynamics, machine learning, high-fidelity modeling, red blood cell

## Abstract

Blood velocity and red blood cell (RBC) distribution profiles in a capillary vessel cross-section in the microcirculation are generally complex and do not follow Poiseuille's parabolic or uniform pattern. Existing imaging techniques used to map large microvascular networks in vivo do not allow a direct measurement of full 3D velocity and RBC concentration profiles, although such information is needed for accurate evaluation of the physiological variables, such as the wall shear stress (WSS) and near-wall cell-free layer (CFL), that play critical roles in blood flow regulation, disease progression, angiogenesis, and hemostasis. Theoretical network flow models, often used for hemodynamic predictions in experimentally acquired images of the microvascular network, cannot provide the full 3D profiles either. In contrast, such information can be readily obtained from high-fidelity computational models that treat blood as a suspension of deformable RBCs. These models, however, are computationally expensive and not feasible for extension to the microvascular network at large spatial scales up to an organ level. To overcome such limitations, here we present machine learning (ML) models that bypass such expensive computations but provide highly accurate and full 3D profiles of the blood velocity, RBC concentration, WSS, and CFL in every vessel in the microvascular network. The ML models, which are based on artificial neural networks and convolution-based U-net models, predict hemodynamic quantities that compare very well against the true data but reduce the prediction time by several orders. This study therefore paves the way for ML to make detailed and accurate hemodynamic predictions in spatially large microvascular networks at an organ-scale.

**Significance Statement**

Existing techniques to image capillary blood vessel networks in vivo do not allow a direct measurement of hemodynamic variables such as the wall shear stress (WSS) that play critical roles in health and disease conditions. Here we present artificial intelligence (AI) techniques that provide highly accurate and fully 3D quantification of blood velocity, red blood cell concentration, WSS, and other critical hemodynamic variables in every vessel in a vascular network. This study paves the way for AI to make hemodynamic predictions in organ-scale capillary vessel networks while retaining the subcellular scale details and overcoming the limitations of the in vivo imaging techniques, with potential applications in hematological and microvascular disorders, angiogenesis, and vascular-mediated drug delivery.

## Introduction

Capillary vessels, the smallest blood vessels in the body, are responsible for delivering oxygen and other metabolites to tissues. Together with vascular bifurcations and mergers, they form a complex network of vessels referred to as the microvascular network (1–3). The distribution of blood flow and red blood cells (RBCs) in the network is critical to the healthy function of the body as it dictates the oxygen and nutrient delivery and waste removal (4, 5). The microvascular network also plays a critical role during vascular remodeling and in diseases, e.g. cardiac and cerebral disorders, diabetes, tumor growth, sickle cell anemia, and malaria. These conditions are known to alter the blood flow and RBC distribution (6–9). A knowledge of the blood flow and RBC distribution in the microvascular network, therefore, is of immense physiological importance.

The blood velocity and RBC concentration profiles over the cross-section of a microvessel are generally complex and established under multiple, and often competing, mechanisms related to RBC deformation and fluid motion in the mosaic-like topology of the microvascular network (10, 11). The velocity profile is not parabolic (i.e. Poiseuille's profile) as is the case for a single-phase fluid flowing in a long, straight tube. The RBC concentration is also nonuniform: Being highly deformable, RBCs undergo a cross-stream migration which tends to increase their concentration near the vessel center and reduce toward the wall, where a cell-*free* layer (CFL) develops (12, 13). The complexity of the profiles increases further in the presence of vascular junctions and vessel tortuosity. Downstream of a vascular bifurcation, the velocity and concentration profiles tend to skew toward opposite sides of a vessel (14–16). The degree of skewness may alter as RBCs flow through subsequent bifurcations (15). Vessel tortuosity also affects the profiles by skewing them toward the side with higher curvature (17, 18).

Obtaining such full, 3D profiles of blood velocity and RBC concentration is important not only for understanding the hemophysics of microvascular flow and for predicting tissue perfusion, but also for an accurate evaluation of critical physiological quantities, such as the wall shear stress (WSS) and CFL. The WSS and its gradient, to which the endothelial cells respond to trigger vasomotion, can be accurately evaluated from the full velocity profile (19–21). The CFL provides a means to reduce the apparent blood viscosity in small vessels as illustrated by the Fahraeus–Lindqvist effect (3, 11–13, 22). A full 3D description of the CFL can be accurately obtained from the corresponding RBC concentration profile (12, 16, 18). The CFL further provides a diffusion barrier to the gas exchange and facilitates platelet and leukocyte margination which are critical to hemostasis and the immune response of the body (3, 11–13).

Although recent advances in imaging techniques in vivo have enabled high-resolution, 3D mapping of the microvascular network at large spatial scales up to an organ level (5, 23–25), measurement of the full, 3D profiles of blood velocity and RBC concentration in every vessel of the network remains difficult (25). Low-dimensional theoretical models of network blood flow are often used to predict vessel-averaged hemodynamic quantities in such experimentally acquired images (26, 27). These models, however, treat each vessel as 1D conduit and assume Poiseuille's law. As such, they cannot provide the full, 3D profiles of the velocity, concentration, WSS, and CFL. In contrast, such detailed information is readily obtained by high-fidelity computational models that retain the three-dimensionality of the vessels and treat blood as a suspension of deformable RBCs. Such models have been used to predict hemodynamics in single microvessels, bifurcations, and physiologically realistic microvascular networks, e.g. Refs. 12, 15, 16, and 28–33. Such models, however, tend to become computationally expensive with the increasing size of the network, therefore, they are not feasible for use in large networks at organ-scale.

To overcome this limitation of high-fidelity models, we consider a machine learning (ML) approach. In recent years, ML techniques have been applied to various microscale hemodynamics studies. Examples include the classification of RBC shapes (34), predicting RBC deformation and trajectory in microfluidic devices (35), estimation of cell deformability (36–38), fast processing of in vivo images (39), and estimating RBC flux in cortical capillary networks (40). ML was also used to integrate images of blood flow with underlying physical laws to infer the flow field in microaneurysm (41).

Recently, our group has developed an ML model to predict blood flow rate and vessel-averaged RBC concentration in the microvascular network (42). This prior model was a spatially 1D model as the velocity and concentration profiles over a vessel cross-section were not considered. In this study, we develop ML models to predict the full 3D blood velocity, RBC concentration, WSS, and CFL profiles in every vessel in the network. Such detailed information can otherwise be obtained only from the high-fidelity models. We demonstrate that the ML predictions compare against the true data with a mean-squared error ≲0.1 but reduce the prediction time by several orders compared to a high-fidelity simulation. This study therefore paves the way for ML to bypass expensive computations and provide highly accurate and full 3D hemodynamic data in spatially large microvascular networks at organ-scale.

## Data generation

**Fig. 1.**
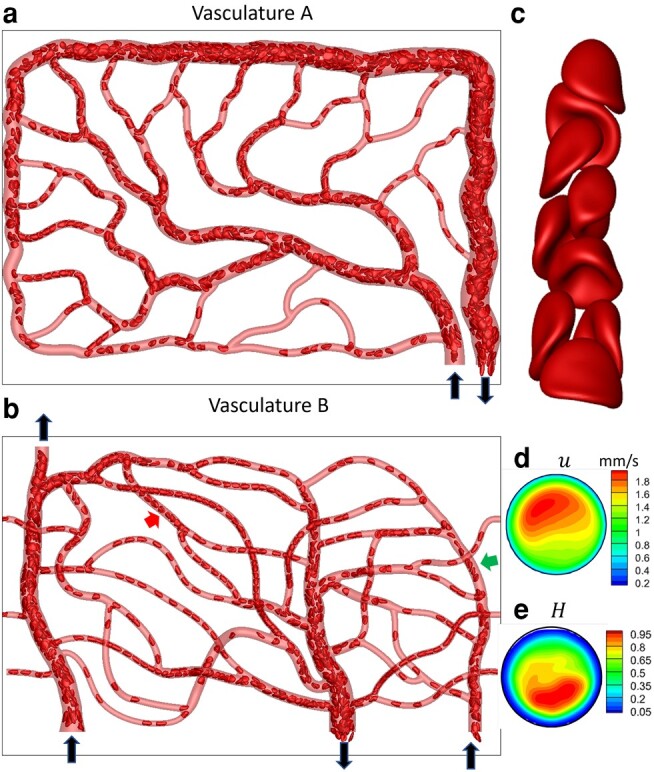
Data generation via high-fidelity RBC-resolved simulations. a and b) A visualization from the simulations for vasculature A and B. Black arrows indicate inlets/outlets. The images are in x,y plane and looking down *z* axis. c) A close-up showing RBC deformed shapes. d and e) Time-averaged velocity and RBC concentration at a vessel cross-section showing nonuniform and highly skewed profiles. Red and green arrows indicate vessels where (c) and (d and e) are sampled.

Our data comes from high-fidelity, 3D simulations of the flow of deformable RBC suspension in two physiologically realistic microvascular networks which are built in silico resembling in vivo images (43). We refer to these networks as vasculatures A and B (Fig. 1); the first is used for training, and the second for testing. Each vasculature is geometrically complex with multiple (∼50) vessels, bifurcations (∼21), and mergers (∼20) and represents a tissue area of ∼0.135 mm^2^. Blood as a suspension of RBCs and plasma flows through the in silico vasculatures, and at any instant of time, there are about 1,000 RBCs present in each. The physical flow time simulated is about 1.5 s, which is more than an average cardiac cycle.

The numerical methodology used in the high-fidelity simulations is based on a coupled finite volume/finite element/immersed-boundary method and is detailed in our previous studies (30, 43). Briefly, the in silico vasculatures are built using CAD software and contained in the computational domain that is discretized by ∼ 160 million mesh points. Fluid motion is governed by the unsteady Stokes equations and continuity equations. A physiologically relevant flow rate is specified as the boundary condition at the network inlets. A ghost-node immersed-boundary method is used to implement the no-slip boundary condition on the vessel surfaces. RBCs are injected at the inlets with an average hematocrit of 30%, and they distribute throughout the vasculature by the flow. Each RBC is modeled as a viscous liquid made of hemoglobin enclosed by a membrane with the undeformed shape of a biconcave discocyte. The membrane is assumed to possess a resistance against shearing, area dilation, and bending. The viscosity difference between hemoglobin and plasma is also taken into consideration. A continuous forcing immersed-boundary method is used to model the two-way coupling between the fluid and RBCs.

A visualization of the RBC distribution in one instant and a close-up of RBC shapes in a vessel segment are shown in Fig. 1a–c. Heterogeneous RBC distribution, which is a hallmark of the microvascular blood flow, is predicted in our simulation. Highly deformed RBC shapes, characterized as parachute and slipper shapes as observed in vivo, are also predicted.

The simulations provide 3D, time-resolved fluid velocity u(x,y,z,t) and RBC concentration H(x,y,z,t) distributions in every vessel in the vasculature. Our specific interest is the time-averaged but spatially varying velocity and concentration profiles defined as $u\equiv u(x,y,z):\;=\int_{T}\;u(x,y,z,t)dt/T$ and $H\equiv H(x,y,z):\;=\underset{T}{\int }\;I(x,y,z,t)dt/T$, respectively, where *T* is the simulation time, x,y,z represents the Eulerian space, and *I* is an indicator function that is one inside a cell and zero outside. The WSS and CFL are readily obtained from u(x,y,z) and H(x,y,z). Three hundred instances of data are used to obtain the averages. We refer to these averages as the direct simulation results or DSR. Figure 1d and e shows u(x,y,z) and H(x,y,z) at one vessel cross-section. As seen, u(x,y,z) and H(x,y,z) are nonuniform, nonparabolic, and highly skewed. The WSS and CFL similarly have complex distributions. Our goal is to develop ML models that can predict such complex spatial distributions.

## ML models and results

Each vascular network is composed of three components: vessels, bifurcations, and mergers. The flow dynamics of RBCs and the mechanisms leading to complex velocity and hematocrit distributions in each vascular component are different. Thus, three separate ML modes are built for each component. Furthermore, the RBC concentration and blood velocity profiles are coupled together due to the coupling between RBC deformation and fluid motion, and hence, they must be predicted simultaneously.

We first build the ML models for each of the three vascular components using the DSR data from vasculature A. Then, we test the models and predict hemodynamic variables in vasculature B in two steps. First, we consider each vascular component in *isolation*: For example, for a bifurcation in vasculature B, we specify the DSR velocity and RBC concentration as the input immediately upstream of the bifurcation and predict the output at the daughter vessels immediately downstream. Next, we consider the entire vasculature-wide prediction. In this, we only specify the DSR data as the input at the inlet of the vasculature and predict the concentration and velocity profiles as they evolve in the entire vasculature by progressing through the hierarchy of vessels, bifurcations, and mergers.

Furthermore, we develop both 2D and 3D models. For the 2D model, the velocity and concentration distributions over the middle *z* plane of the network are considered so that u=u(x,y) and H=H(x,y) (see Fig. 1a and b). The advantage of 2D models is that they are less complex and require less amount of training data. For this, we use the artificial neural network (ANN). Thereafter, we consider a 3D model to predict u(x,y,z) and H(x,y,z) for which we use a convolution neural network-based U-net model.

### 2D ML models

Bifurcations and Vessels and Mergers sections describe ML models for vascular components in isolation, and Vasculature-Wide Prediction section for the whole vasculature-wide prediction.

#### Bifurcations

**Fig. 2.**
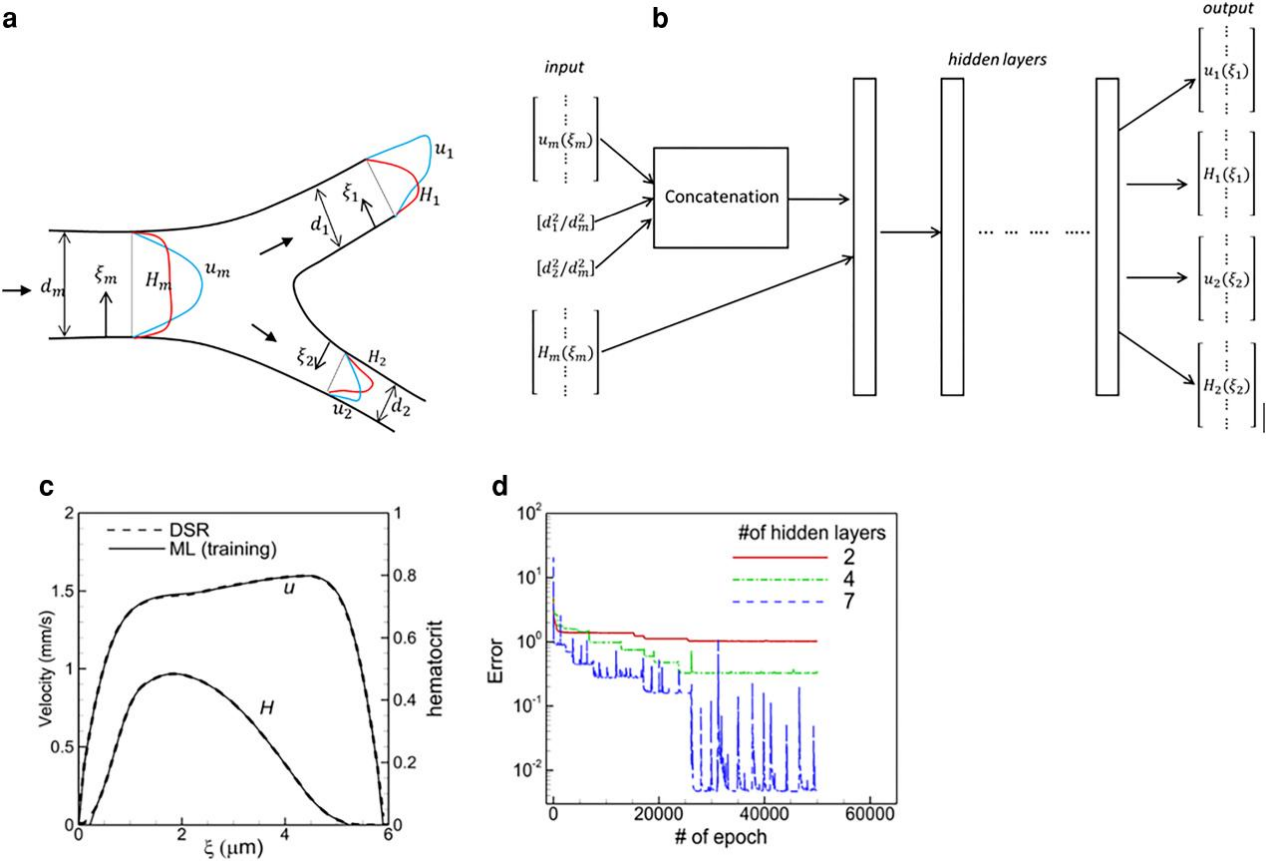
a) Schematic of the ML model and b) the structure of the ANN for a bifurcation. Notations are defined in the text. c) Comparison of the DSR and ML in the training process in a daughter vessel of one bifurcation in vasculature A. d) Convergence of training error for different numbers of hidden layers.

The goal here is to predict the velocity and RBC concentration profiles in the daughter branches downstream of a bifurcation, namely, $u_{d1}(\xi_{1}),\;H_{d1}(\xi_{1}),\;u_{d2}(\xi_{2}),\;H_{d2}(\xi_{2})$ when the corresponding conditions in the mother vessel, $u_{m}(\xi_{m}),\;H_{m}(\xi_{m})$, are known, where $\xi_{1},\;\xi_{2},\;\xi_{m}$ indicate, respectively, a local coordinate along the diameters of the daughter and mother vessels (Fig. 2a). As RBCs flow through a bifurcation their concentration becomes biased toward the side of a daughter vessel that is closer to the apex of the bifurcation, while the velocity profile is biased to the opposite side. With respect to a global coordinate, each profile is thus oppositely biased in the two daughter vessels. To appropriately include such bias, $\xi_{1}$ and $\xi_{2}$ should be globally opposite but locally from the similar (e.g. apex side) side in each daughter vessel.

The DSR data is first prepared to generate the input and output vectors for training as $\left[u_{m_{1}},u_{m_{2}}\;..\;.\;\ldots \;u_{m_{i}}\ldots \ldots \;u_{m_{N}}\right]$, $\left[H_{m_{1}},H_{m_{2}}\;..\;.\;\ldots \;H_{m_{i}}\ldots \ldots \;H_{m_{N}}\right]$, $\left[u_{d1_{1}},u_{d1_{2}}\;..\;.\;\ldots \;u_{d1_{i}}\ldots \ldots \;u_{d1_{N}}\right]$, etc., where i=1,…..N represents the collocation points. Since the number of finite volume mesh points differs between vessels of different diameters, each vessel's DSR data were interpolated to vectors of length N=56 via cubic spline interpolation. Also, the data is smoothened using spline smoothing to have a well-converged model.

Additional input features required for the ML model are the ratio of the daughter to mother vessel cross-sectional area, i.e. $(d_{1}/d_{m}{)}^{2}$ and $(d_{2}/d_{m}{)}^{2}$.

Figure 2b shows the ANN structure for the bifurcation. The inputs $(d_{1}/d_{m}{)}^{2}$, $(d_{2}/d_{m}{)}^{2},\;u_{m_{i}}$ and $H_{m_{i}}$, i=1,…N, are passed through several layers. A concatenation is required to convert the scalar inputs into vectors as $\left[{\left(d_{1}/d_{m}\right)}^{2}\;\;\;{\left(d_{1}/d_{m}\right)}^{2}\;\;\;{\left(d_{1}/d_{m}\right)}^{2}\;\ldots \ldots \ldots \;\right]$ and $\left[{\left(d_{2}/d_{m}\right)}^{2}\;\;\;{\left(d_{2}/d_{m}\right)}^{2}\;\;\;{\left(d_{2}/d_{m}\right)}^{2}\;\ldots \ldots \ldots \;\right]$ and stack them with $u_{m_{i}}$. The concatenated input is then passed to the next layer to combine with $H_{m_{i}}$. We found that a concatenation done before the first hidden layer yields the best result, since there is no transformation of the velocity. The velocity and diameter squared give the information about volume flow rate which is preserved, and hence provides a better correlation between input and output. However, this is not the case if we concatenate in other layers where velocity is transformed. Also, since each of the four inputs represents separate physical variables, they must be passed as four different vectors, instead of combining into one vector.

A single ML model is developed using the data from all bifurcations in vasculature A. The training process starts with a randomly selected bifurcation and the loss function using mean squared error (MSE $=\sqrt{\overset{N}{\underset{i=1}{\sum }}\;{\left(u_{i}^{\left(\mathrm{DSR}\right)}-u_{i}^{\left(\mathrm{ML}\right)}\right)}^{2}/N}$ for velocity and $\sqrt{\overset{N}{\underset{i=1}{\sum }}\;{\left(H_{i}^{\left(\mathrm{DSR}\right)}-H_{i}^{\left(\mathrm{ML}\right)}\right)}^{2}/(N\cdot \max\{H_{i}^{2}\}}$*)* is minimized. The process is repeated for all bifurcations resulting in one epoch. The training ends when the error is sufficiently reduced after several epochs. Numerical experiments were performed by varying different hyperparameters to obtain the best prediction. Figure 2d shows the error for various numbers of hidden layers. The final model has seven hidden layers each with 80 neurons, the Adam optimizer with a learning rate of $10^{-3}$, and the rectified linear activation function (ReLU). A dropout layer is implemented after each hidden layer to prevent overfitting. Without this, the error in prediction can be large even though the error in training is small. Figure 2c compares the DSR data and the training result, demonstrating high accuracy of training.

**Fig. 3.**
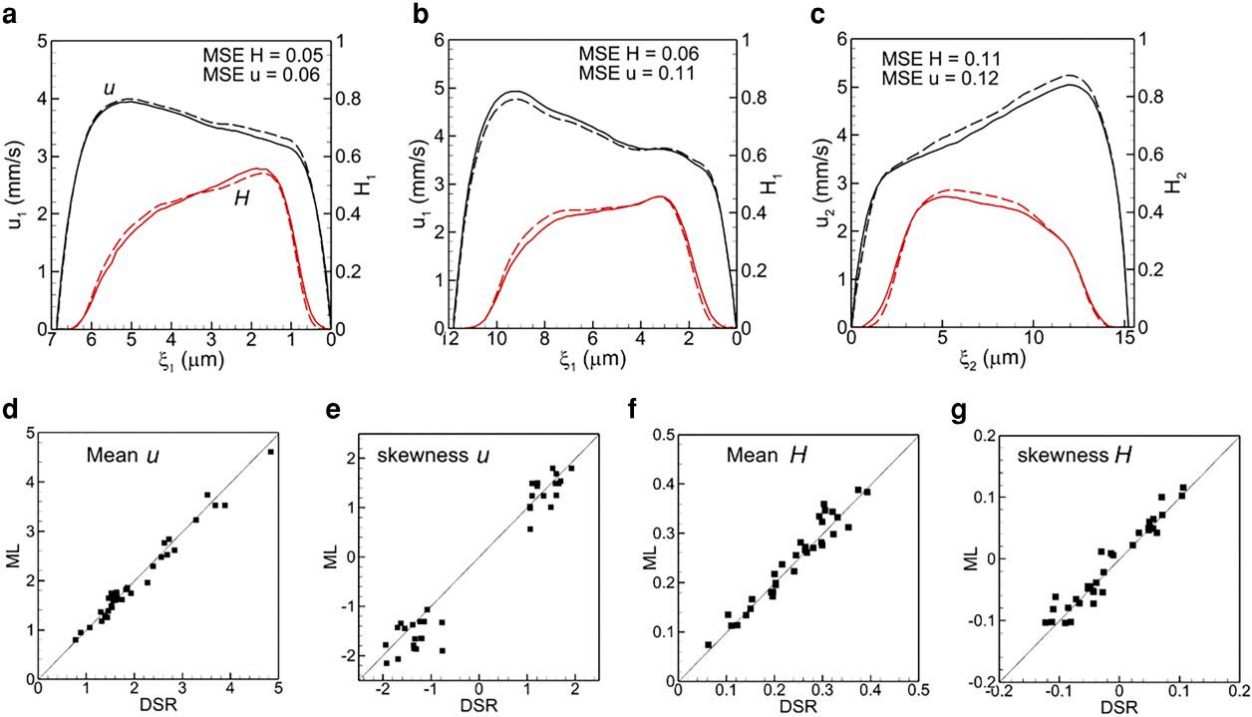
a–c) comparison of ML prediction and DSR data for three isolated bifurcations in 2d. Black curves are for *u*, and red for *H*. Dash curves represent ML and solid curves represent DSR. The mean and skewness of *u* and *H* profiles are compared in (d–g).

Once the ML model is trained, we test it for each isolated bifurcation of vasculature B. For this, the DSR data in the mother vessel is used as the input. The model then predicts *u* and *H* profiles in the two daughter vessels as the output. Figure 3 shows a comparison of the ML-predicted profiles against the DSR data in three selected bifurcations. The agreement between them is excellent. An additional comparison is given in Fig. S2. The average MSE of the ML prediction is ∼0.1 for *u* and ∼0.08 for *H*. In relative to typical average values, this amounts to ∼4−5% error in *u* and ∼10% error in *H*. Figure 3 also compares the mean and skewness of *u* and *H* for all bifurcations as obtained in the DSR and predicted by the ML, which also shows good agreement between the two.

#### Vessels and mergers

**Fig. 4.**
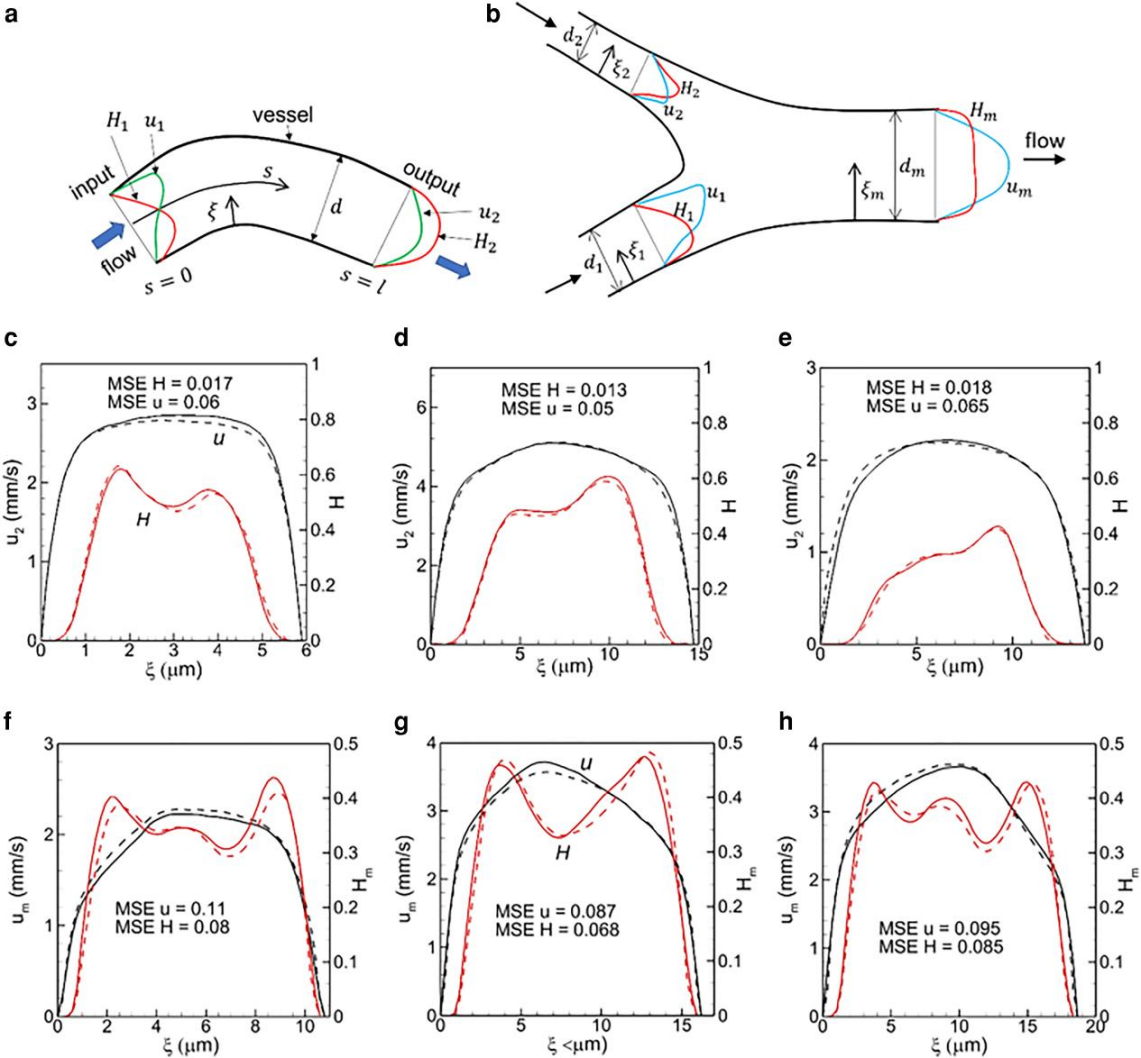
Schematic of the ML model for (a) vessels and (b) mergers. c–e) Predictions for vessels, and f–h) for mergers. Black represents velocity, and red hematocrit. Continuous curves are DSR data, and dash curves are ML prediction.

Next, we build ML models for the remaining two vascular components, i.e. vessels and mergers. For vessels, the inputs are the velocity and concentration $u_{1}(\xi )$ and $H_{1}(\xi )$ at an upstream location, vessel diameter *d*, and length *l*; the outputs are $u_{2}(\xi )$ and $H_{2}(\xi )$ at a downstream location (Fig. 4a). For mergers, inputs are $u_{1}(\xi )$, $H_{1}(\xi )$, $u_{2}(\xi )$, $H_{2}(\xi )$ in upstream vessels, and area ratios $(d_{1}/d_{m}{)}^{2}$ and $(d_{2}/d_{m}{)}^{2}$; the outputs are $u_{m}(\xi )$ and $H_{m}(\xi )$ in the downstream merged vessel (Fig. 4b). Note that in the current model, outputs are predicted only at the end of the vessel length *l*, not at the intermediate locations s<l. If predictions are needed at intermediate locations, the model needs to be modified. The ANN structures are given in Fig. S3. The scalar inputs are concatenated with velocity and then passed to the first hidden layer along with hematocrit. The similar hyperparameters as in the previous section are used except for 10 hidden layers for the merger model. One ML model is built using the data from all vessels in vasculature A, and another for mergers. Similar convergence as in the previous section yielding high training accuracy is also achieved.

Figure 4c–e shows ML-predicted $u_{2}$ and $H_{2}$ in three vessels in isolation from vasculature B. Results for mergers are shown in Fig. 4f–h. Additional data are given in Figs. S4 and S5. For vessels, the average MSE is ∼ 0.1 for *u* and 0.07 for *H*; for mergers, they are ∼ 0.1 and 0.08, respectively, suggesting good agreement between the DSR and ML.

#### Vasculature-wide prediction

**Fig. 5.**
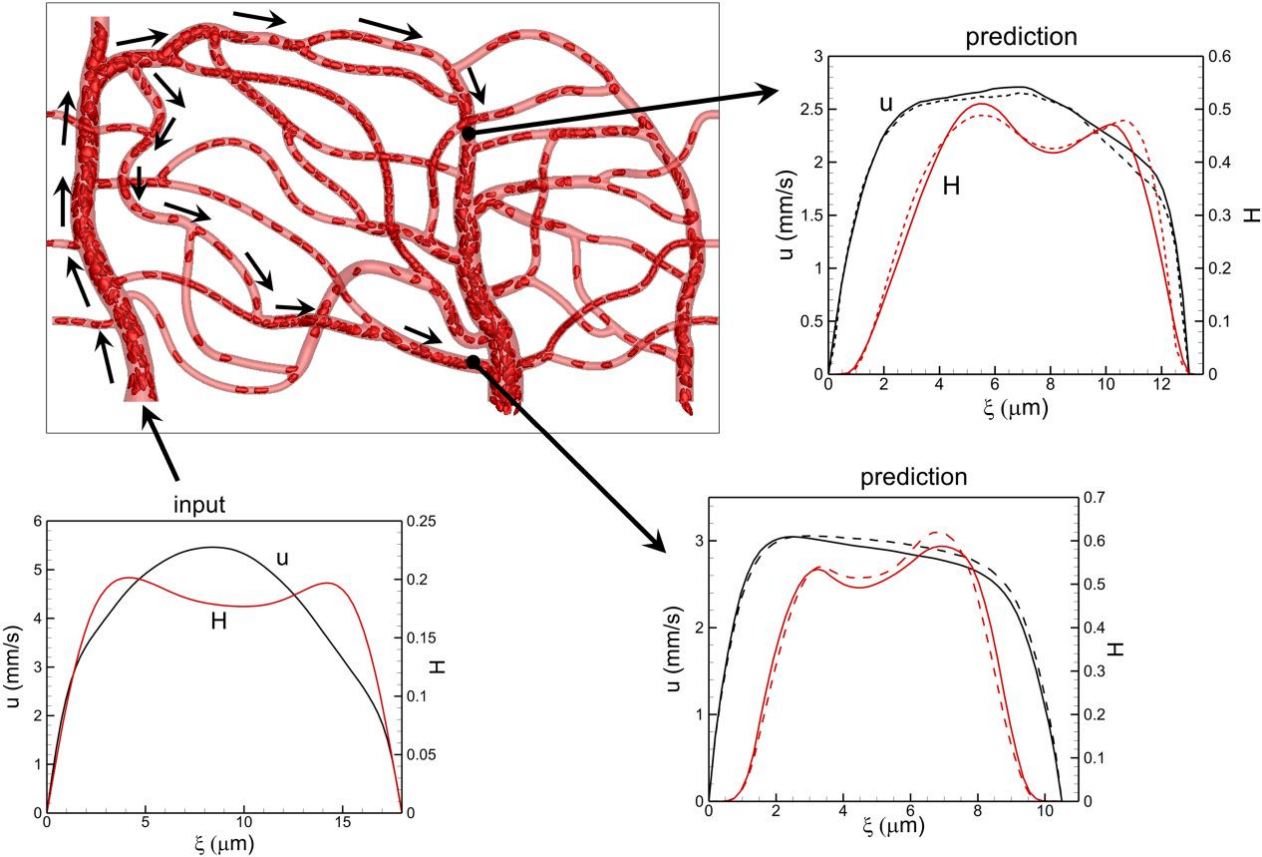
Vasculature-wide 2D prediction following two different paths shown by arrows. ML and DSR results are compared at the end of the paths. Black and red colors represent *u* and *H*, respectively. Solid and dash curves represent DSR and ML prediction, respectively.

We now test the models to make vasculature-wide predictions. For this, the DSR data is specified as input only at the vasculature inlet, and predictions are made sequentially through the hierarchy of the bifurcations, vessels, and mergers, with the prediction from one vascular component used as the input to the next component. Figure 5 compares the ML and DSR data at the end of two different paths that include multiple vascular components. Predictions along additional paths are given in Fig. S6. The MSE based on all vascular components in each path predicted is ∼  *0.07—0.1* for *u* and *H*, suggesting good agreement between the DSR and ML.

### 3D ML models

**Fig. 6.**
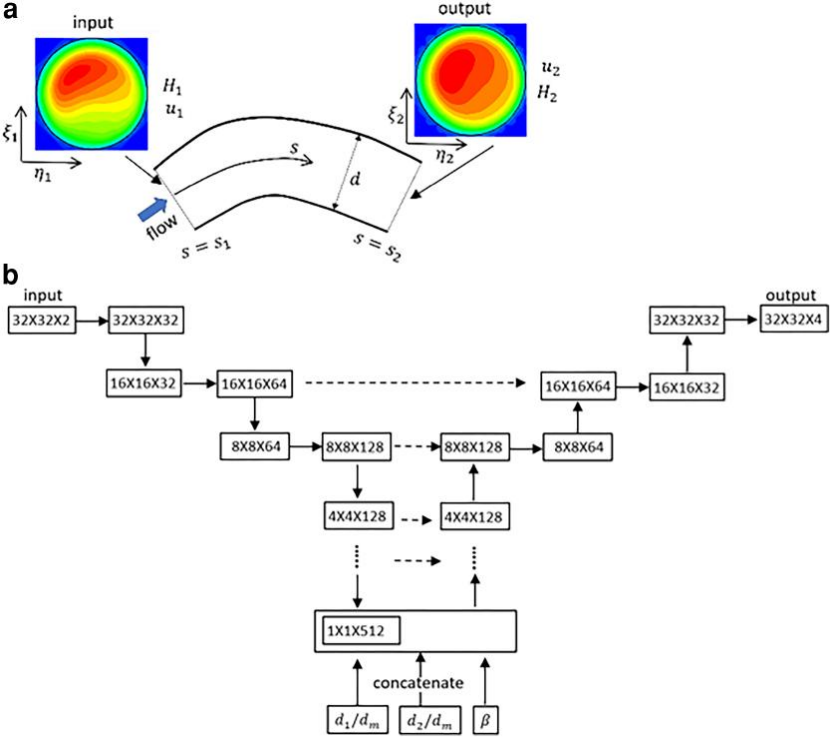
a) Schematic of 3D prediction. b) U-net model for a bifurcation. The left-hand side is the contraction (encoder) path, and the right side is the expansion (decoder) path. Each horizontal continuous arrow represents two consecutive convolutions, downward arrow represents max-pooling and upward arrow represents transposed convolutions. The horizontal dashed arrow represents concatenation of the output of the transposed convolution layers with the feature maps from the encoder.

The 3D problem is schematically shown in Fig. 6a. Given $u_{1}(\xi_{1},\eta_{1})$  :=  $u(s=s_{1},\xi_{1},\eta_{1})$ and $H_{1}(\xi_{1},\eta_{1}):\;=\;H(s=s_{1},\xi_{1},\eta_{1})$, we seek to predict $u_{2}(\xi_{2},\eta_{2}):\;=\;u(s=s_{2},\xi_{2},\eta_{2})$ and $H_{2}(\xi_{2},\eta_{2}):\;=\;$$H(s=s_{2},\xi_{2},\eta_{2})$, where s represents the streamwise coordinate, $s_{1}$ and $s_{2}$ are upstream and downstream locations, respectively, and $\xi_{1},\eta_{1}$ and $\xi_{2},\eta_{2}$ are *local* coordinates over the vessel cross-section at those locations. As such, we now deal with data arranged in an N×N matrix, where *N* is the number of collocation points in each direction. A vector-like (i.e. flattened) arrangement of the data will cause a loss of correlation in one spatial direction. We therefore consider a *U-net model* which is one form of the convolution neural networks (CNN) having both the feature extraction through a contracting path (also called down-sampling or encoder) and feature addition through an expanding path (i.e. decoder or up-sampling).

Through numerical experiments, we found that at least N=32 is needed to capture the spatial variation of *u* and *H*. A data augmentation is done by incrementally rotating the DSR data by a small, arbitrary angle Δβ, with each rotation giving a additional data. For each vascular component, about 100 such additional data are created. Also, the data from two bifurcations in vasculature B are used to further augment the training data since 3D predictions require more data due to increased size (N×N) of the variables.

The U-net structure is shown in Fig. 6b for bifurcations. The encoder path is made of a sequential application of two consecutive regular convolutions using trainable filters of size 3×3 followed by a max-pooling which extracts the maximum value associated with a feature and reduces the size of the data. The down-sampling process continues until the data is flattened. Then a concatenation is performed to include geometric parameters, e.g. $d_{1}/d_{m}$, $d_{2}/d_{m}$, and Δβ. Note that the velocity and concentration profiles are biased in specific manner relative to the geometry of a bifurcation. If the data is rotated, this relative orientation (Δβ) with respect to the bifurcation geometry must be provided. The up-sampling path is composed of transposed convolutions and a regular convolution. Additionally, at each up-sampling layer, the feature maps from the down-sampling layer are concatenated.

Three U-net models corresponding to each vascular component are built. The nonlinear ReLU activation is used for all. A constant learning rate of $5\times 10^{-4}$ and $6\times 10^{4}$ epochs is used for bifurcations and vessels. For mergers, variable learning rates and a higher number of epochs are used as follows: $5\times 10^{-4}$ for the first $5\times 10^{4}$ number of epochs, followed by $10^{-4}$ for the next $3\times 10^{4}$, and $5\times 10^{-5}$ for the final $3\times 10^{4}$ epochs. These rates are determined by numerical experiments and only applied to the training process, and hence using the training data only.

**Fig. 7.**
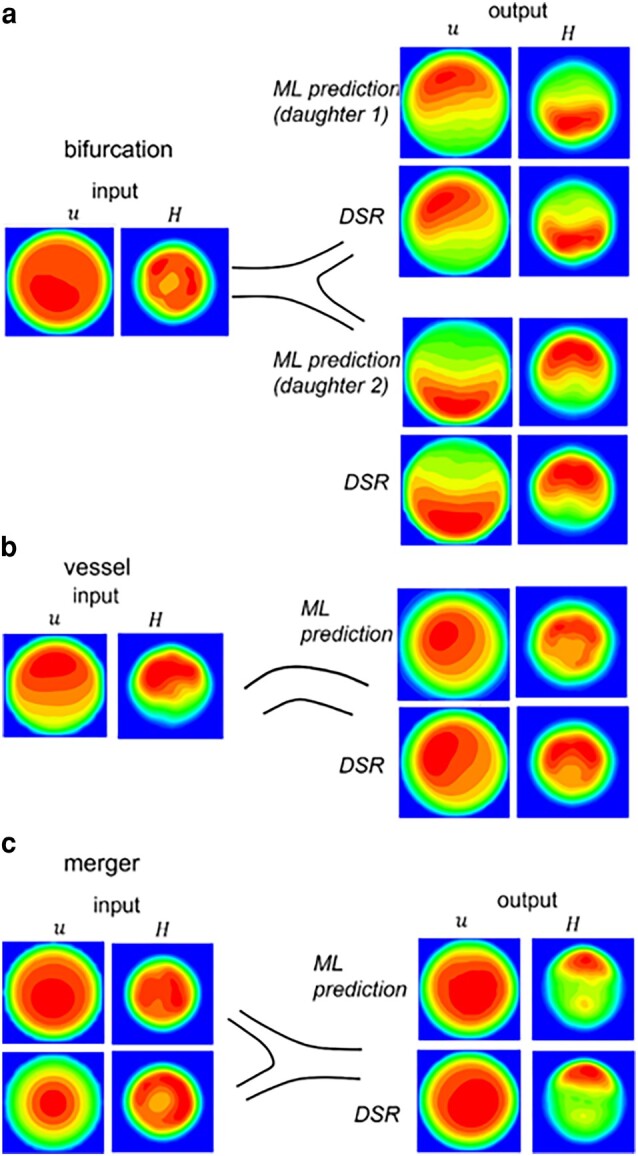
3D ML prediction in isolated bifurcations, vessels, and mergers. The left column is *u* and *H* at an upstream location used as input to the model. The right column is the output. The ML prediction is compared against the DSR data.

Figure 7 compares the ML prediction and DSR data for individual vessels, bifurcations, and mergers in isolation. Additional data are in Figs. S7–S9. The 3D predictions compare well against the DSR as the average of the mean absolute error (MAE) is ∼0.09.

**Fig. 8.**
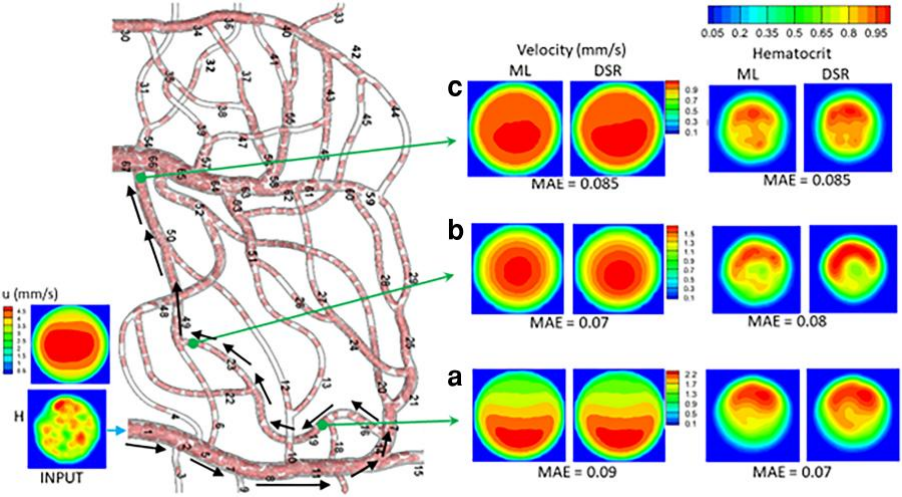
Vasculature-wide 3D prediction. Black arrows indicate the path. ML and DSR results are compared at three locations as marked by green arrows.

Figure 8 shows the vasculature-wide prediction using the 3D model. The ML and DSR results are compared at three locations along a selected path. Results for additional paths are given in Figs. S10–S12. In all cases, the MAE is 0.07—0.12 for u and 0.07—0.1 for *H*, suggesting a good agreement.

### WSS and CFL

**Fig. 9.**
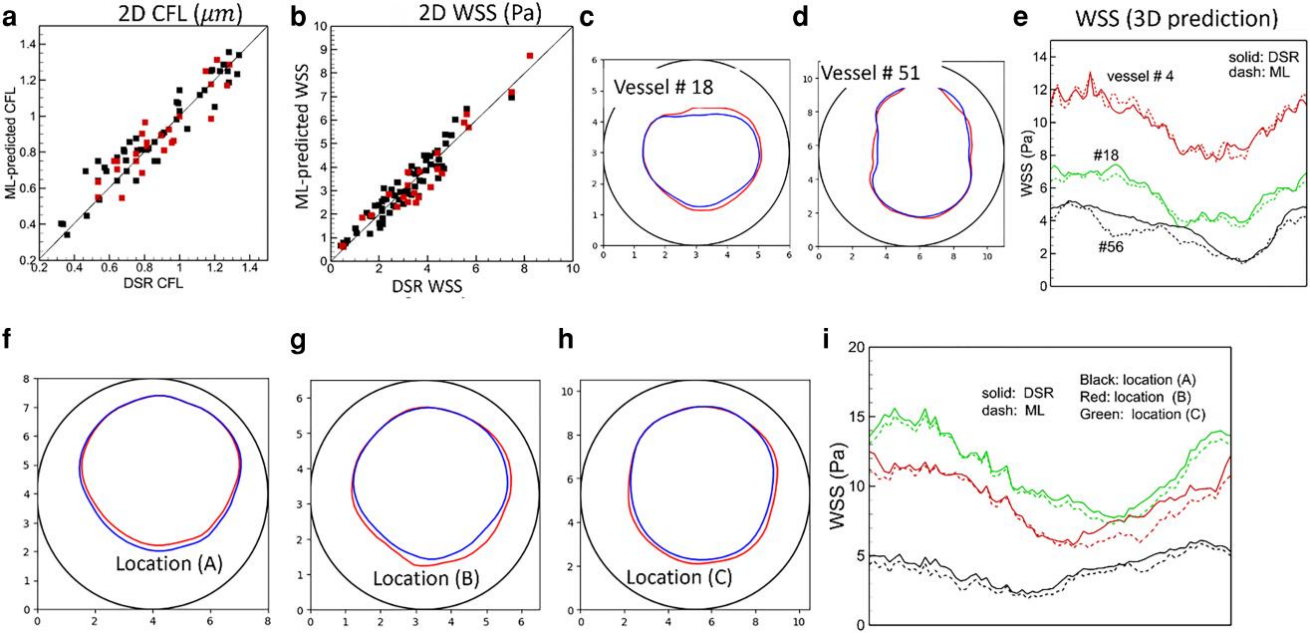
CFL and WSS prediction. a and b) 2D model. Black symbols: individual vascular component; red: vasculature-wide prediction along the paths shown in Fig. 5. c–e) 3D model for individual vascular components. f–i) 3D model for vasculature-wide prediction following the path shown in Fig. 8. The CFL and WSS are shown at three locations (A, B, C) as in that figure. In (c), (d) and (f)–(h) the interface between the plasm layer and RBC-core is shown in color (blue: DSR, red: ML), and the vessel boundary is indicated by black circle. The WSS in 3D prediction is shown along the circumference (horizontal axis in the plot) of the vessel. Numbering of the vessels is given in Figs. 8 and S1.

The WSS and CFL can be obtained from ML-predicted *u* and *H* and compared against the DSR data for each vascular component in isolation and for the vasculature-wide prediction, both in 2D and 3D (Figs. 9 and S10–S14). The WSS is obtained as the product of the radial gradient of *u* and plasma viscosity since the numerical stencil used to calculate the radial gradient is inside the near-wall CFL (21). The CFL is the distance from the wall where H≈0.085, which delineates the interface between the CFL and cell-rich core. This threshold value is decided based on a direct evaluation of the CFL using RBC data (18). Before computing the WSS and CFL, the ML-predicted *u* and *H* are filtered to remove small-amplitude noise. Our results show that the ML predictions compare well against the DSR data.

### Timing comparison

In terms of computation time, the high-fidelity simulation of each vasculature took about 75,000 core-hours (wall-clock time × number of cores) on Intel Xeon Gold 6230 (Cascade Lake) CPUs to simulate one second of blood flow. In contrast, the training of the ML models took on average about seven hours on NVIDIA Tesla T4 GPU, and the predictions took only a few seconds. Additional few hours were also needed for data preparation. The ML models, therefore, reduce the time for hemodynamic prediction by several orders.

## Discussions and conclusions

Existing imaging techniques of capillary vessel networks in vivo do not allow direct measurements of blood velocity and RBC concentration profiles over each vessel cross-section. Such information is needed to obtain physiologically important hemodynamic variables such as the WSS and CFL. High-fidelity, RBC-resolved simulations can provide such details, but they are computationally expensive. Here we presented ML models that can bypass such expensive computations but predict blood velocity and RBC concentration profiles in every vessel in a network. To train and test the models, we acquire data from high-fidelity simulations of deformable RBC suspension flowing in physiologically realistic in silico microvascular networks. A regression-type ANN model is used for 2D prediction, and a convolution-based U-net model is used for 3D. The models are first tested for individual vessels, bifurcations, and mergers. Thereafter, vasculature-wide predictions following different flow paths that involve multiple vascular components are considered. ML predictions compare against the high-fidelity simulation data with ∼0.1 MSE, correctly predicting the highly nonuniform, skewed profiles of the blood velocity and RBC concentration.

The time taken by the ML models to predict the hemodynamic quantities in the networks considered here is found to be several orders less than that of the high-fidelity model. Indeed, the high-fidelity models are based on fundamental principles, provide a large amount of information, and reveal new physics. In many applications, only a few, specific hemodynamic variables may be of interest and the discovery of new physics is not intended. An example is the WSS distribution in a network which is often the intended hemodynamic variable. In such situations, the high-fidelity model can be avoided, and the ML models, instead, can be used to provide highly accurate, detailed data. The high-fidelity models also require high-performance computing resources and specific expertise of the user, whereas the ML models can be run on web-based platforms and by users with wider domain expertise.

The vasculatures used here span over relatively smaller tissue regions compared to what current in vivo imaging techniques can map at an organ-scale, e.g. the human retina (5), and whole mouse brain (23). Detailed and accurate hemodynamic quantities in such massive networks would be useful, for example in understanding the progression of retinopathy, Alzheimer's disease, and dementia, but cannot be feasibly obtained from high-fidelity simulations. In contrast, the significant reduction in the prediction time makes the ML models highly viable for this.

Although the present ML models are trained and tested using simulated RBC flow in microvasculature in silico, they can also be used for predictions using in vivo images and experimental data. Since simultaneously imaging the vasculature and measuring the profiles of RBC concentration and blood velocity is not possible, the ML models presented here can be an effective tool that can accurately predict detailed, 3D hemodynamic parameters in every vessel of the in vivo networks. Remarkably, using the current ML “bank”, the models can be used to predict hemodynamics in the entire vasculature that could consist of a large number of vessels and vascular junctions. It also implies that in complex vascular topologies, for which some hemodynamic information may be missing, the ML model can be applied to fill such voids. Also, the approach is generalizable to multiple inlets as is the case for the testing vasculature used here. If several inlets act as arteries, the vessel and bifurcation models can be applied sequentially to each of them. If several of the inlets are veins, the vessel and merger models can also be applied sequentially.

As in any ML application, the error reduces with an increasing amount of training data. The amount of data used here is deemed to be modest. Furthermore, the error depends on how closely the training and testing vasculatures match in terms of both their geometry and controlling hemodynamic parameters, such as flow rate and vessel hematocrit. The distribution of vessel diameter over successive generations generally follows Horton's law (2) which provides some sort of commonality of the topology in two vasculatures. However, when compared at the level of individual vascular components, there are differences between the two networks. These competing factors resulted in varying accuracy between different vessels. The availability of additional training data spanning a larger parameter space, both in terms of geometry and controlling flow parameters, will reduce the error. Furthermore, for the vasculature-wide prediction, continuous growth of error is not observed, implying that a limited number of trained models can be used for predictions in large networks. If the geometry and flow conditions are very different in two networks, then the error will be high. Also, both vasculatures have similar number of vascular components and span over similar tissue area. If the testing vasculature has a lot more vascular components that differ from the training vasculature, the error is expected to grow.

Some limitations of the current ML models can be noted. The vessels considered here are cylindrical and have constant diameter. As such, the ML model as presented here is not applicable to noncircular vessels and those with changing diameters both of which can affect H (44). Also, the vessel curvature effect is not considered. However, these additional features can be considered in future models by introducing appropriate geometric parameters as additional inputs. Also, the ML models are not vasculature-specific; vasculatures can be swapped for training and testing (42). Furthermore, the model inputs are local concentration and velocity profiles for each vascular component; so even though the DSR data is obtained for a fixed inflow hematocrit and flow rate, the model is not limited to this. The current model, however, is limited to time-averaged hemodynamics. Time-dependent quantities, e.g. WSS fluctuations cannot be predicted by this model. Additionally, the DSR data are generated on a healthy vasculature under normal conditions with regular vascular geometry; as such the ML models developed here can only be used under such conditions. To enable predictions for unhealthy conditions and transient changes in specific vessel conditions, one must first generate the relevant data. Modifications to the model may also be necessary to represent abnormal conditions.

The current ML models are not physics-informed models. No explicit physical constraint was imposed. The model tries to learn the physics from the DSR data which obeys the conservation laws. The models are constructed such that the inputs, e.g. *u*, *H*, and *d*, enable to preserve the flow rate and RBC flux. We did not see any loss in such conserved quantities except one or two bifurcations which have very different geometry and flow conditions compared to the training vasculature.

The current ML models, which to our knowledge are the first of their kind, are highly promising for image-based predictions of subcellular resolved capillary hemodynamics in organ-scale networks (24, 26). ML models following the same techniques presented here can be built for predicting hemodynamics in blood cell disorders, such as sickle cell anemia, malaria, and diabetes mellitus, that are characterized by reduced RBC deformability (6, 7, 45, 46). They can also be used to predict transport of biomolecules and drugs in diseased vasculature, e.g. tumor microvasculature (8, 9). Further extensions can predict altered hemodynamics during vascular adaptation, e.g. during embryonic development, angiogenesis, vasculopathy, and cerebrovascular dysfunction. They can also be applied to nonbiological applications, such as fluids and tracer transport in porous media resolved with pore-scale details.

## Supplementary Material

pgae043_Supplementary_Data

## Data Availability

Data, code, and documentation are available in Github and can be downloaded from https://github.com/SamEbiRutgers/ML_Microvasculature.git.
