# Supplementary material for: Predicting capillary vessel network hemodynamics in silico by machine learning
Source: PNAS Nexus. 2024 Jan 31;3(2):pgae043. doi: 10.1093/pnasnexus/pgae043 (PMC11079571; doi:10.1093/pnasnexus/pgae043)
Supplement: pgae043_Supplementary_Data [file pgae043_supplementary_data.pdf]

Supplementary Materials

Predicting capillary vessel network hemodynamics *in silico* by machine learning

Saman Ebrahimi and Prosenjit Bagchi[#]

Mechanical and Aerospace Engineering Department

Rutgers, The State University of New Jersey

Piscataway, NJ 08854

[#] Corresponding author. E-mail: pbagchi@soe.rutgers.edu

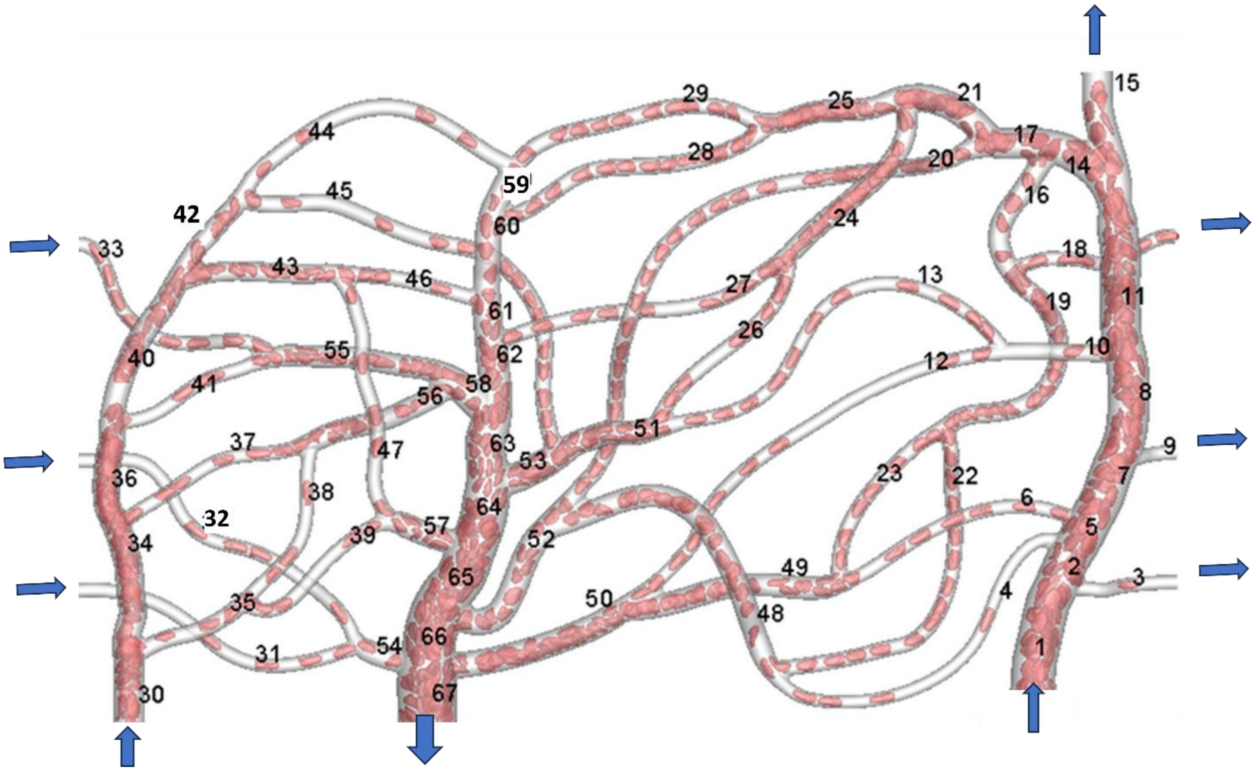

Fig. S1. Numbering of vessels for vasculature 2. Arrows indicate flow directions.

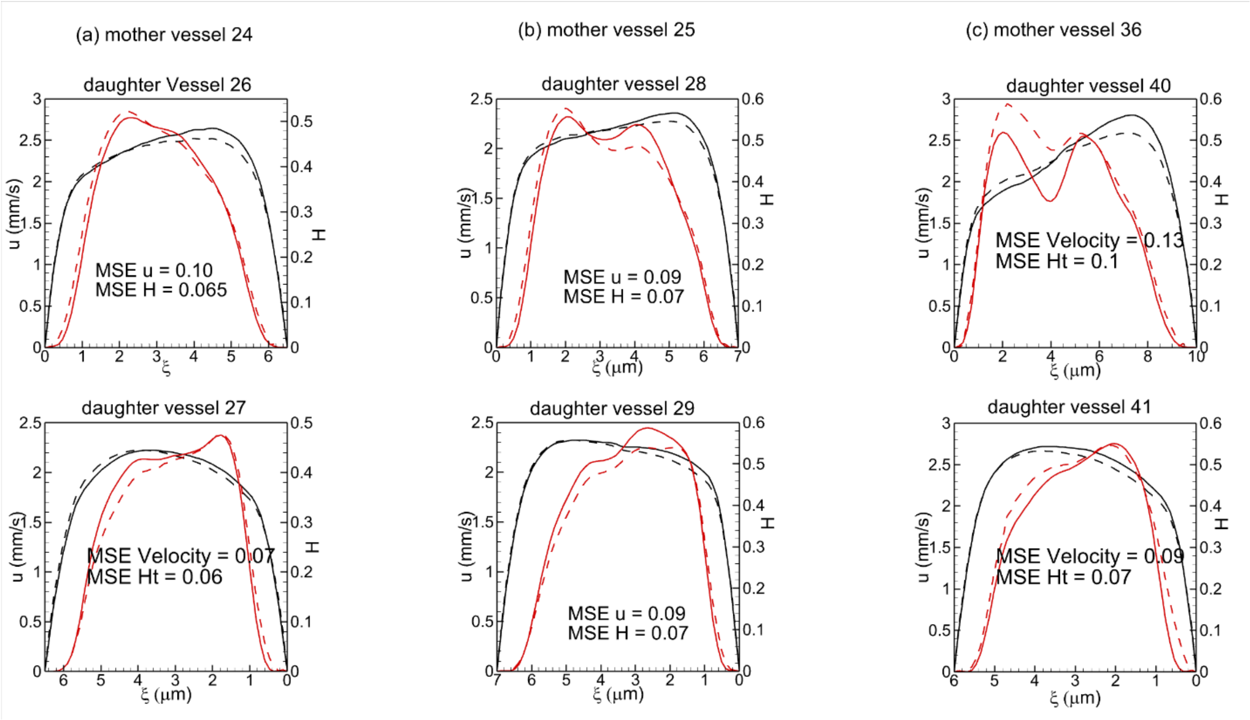

Fig. S2. Additional data for 2D prediction in individual bifurcation. (a)-(c) show three examples. Black and red lines represent velocity and hematocrit, respectively. Continuous and dash lines represent DSR and ML results, respectively. Vessel numbering is shown in Fig. S1.

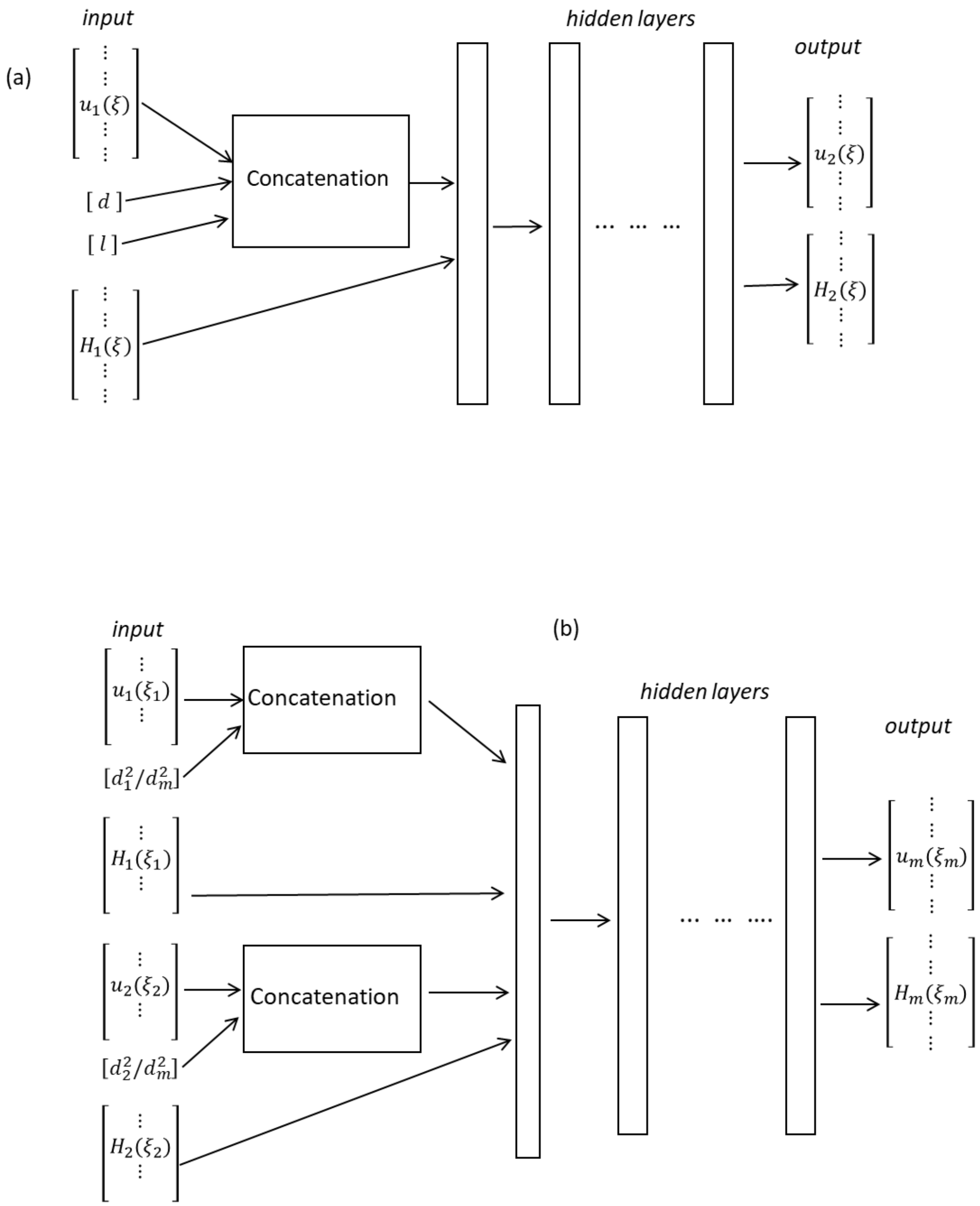

Fig. S3. ANN structure for (a) vessels and (b) mergers.

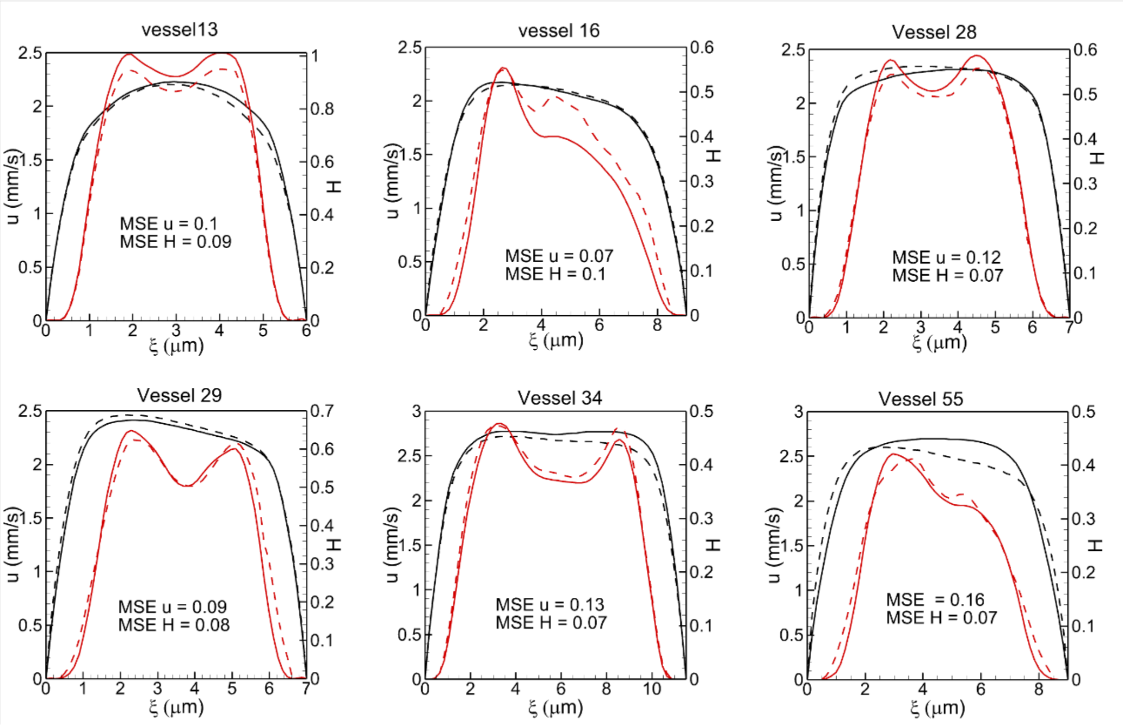

Fig. S4. Additional data for 2D prediction in individual vessels. Symbols are similar as in Fig. S2.

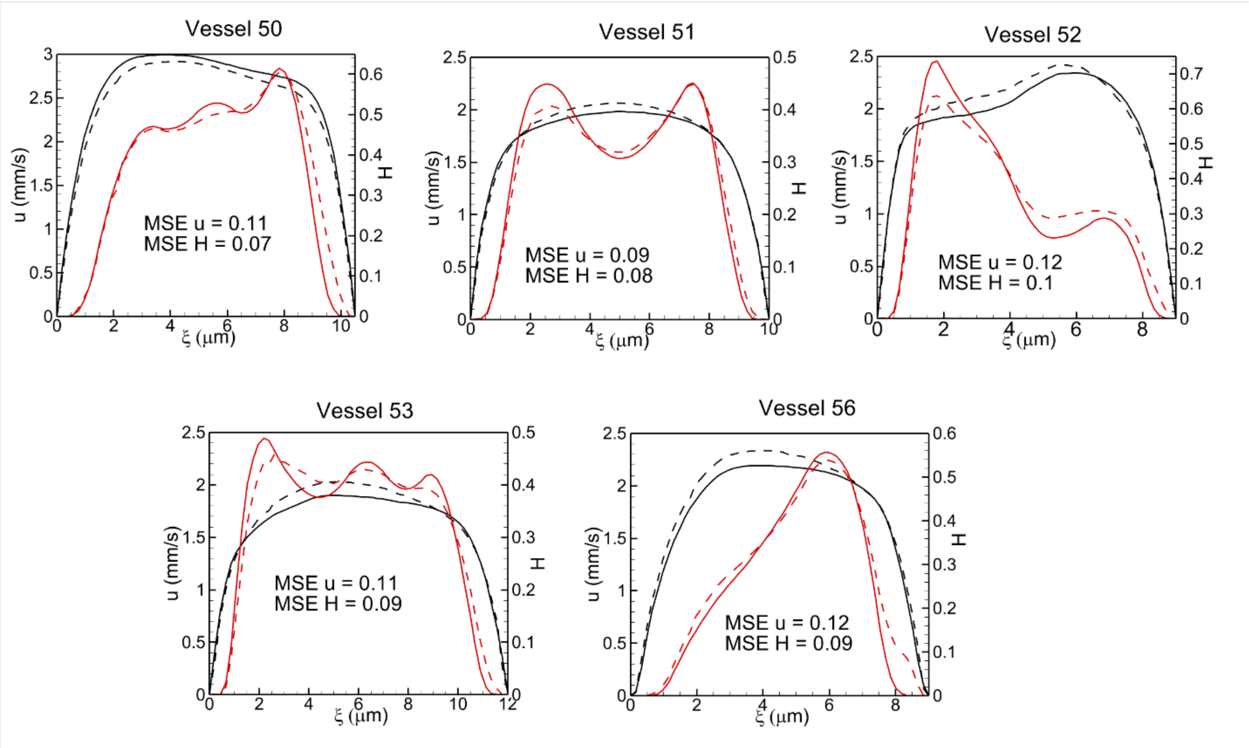

Fig. S5. Additional results for 2D prediction in individual mergers. Symbols are similar as in Fig. S2.

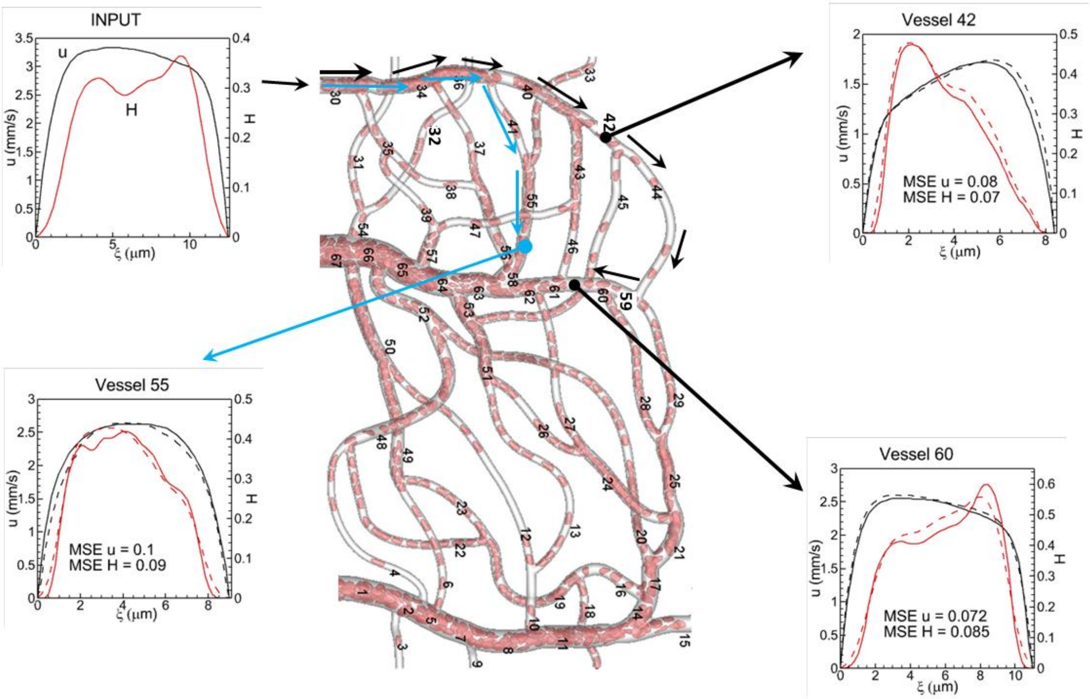

Fig. S6. Additional results for vasculature-wide 2D prediction following two paths shown by black and blue arrows. Input is given at one inlet of the vasculature and ML predictions are made moving along the arrow direction with output from one vascular component fed as input to the next. DSR and ML are compared at three locations. The colors and line patters are similar as in Fig. S2.

Mother vessel #	Daughter vessel #s	DSR velocity (mm/s)	ML velocity	DSR hematocrit	ML hematocrit	MAE
5	7	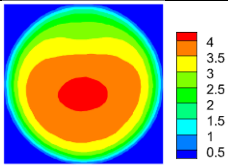	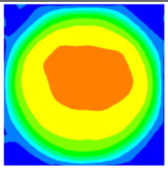	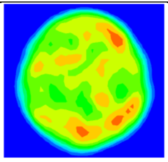	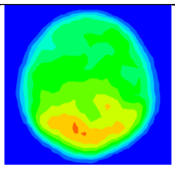	u: 0.15<br>H: 0.13
	6	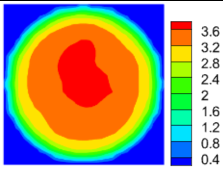	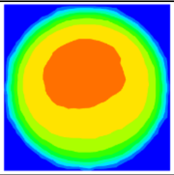	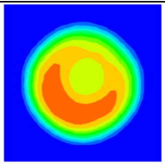	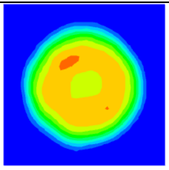	U: 0.12<br>H:0.078
16	18	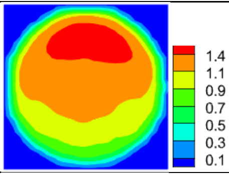	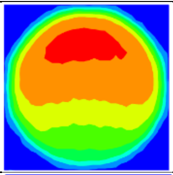	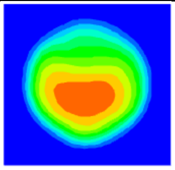	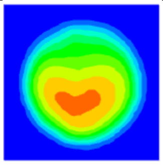	U: 0.11<br>H: 0.092
	19	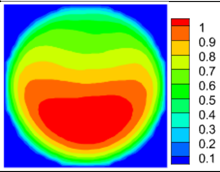	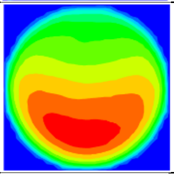	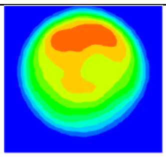	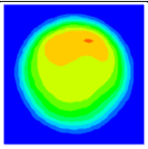	U: 0.084<br>H: 0.066
19	22	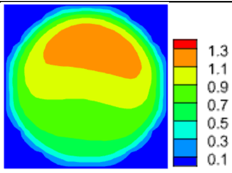	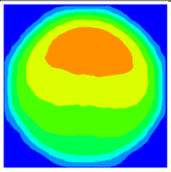	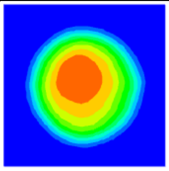	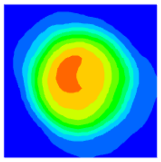	U: 0.089<br>H: 0.083
	23	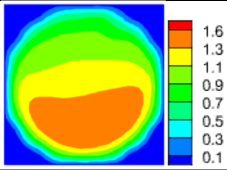	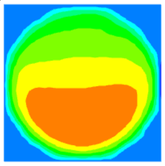	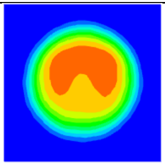	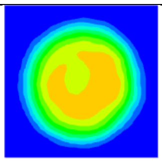	U: 0.075<br>H: 0.1
24	26	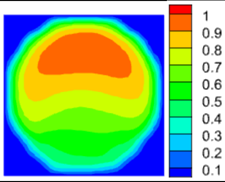	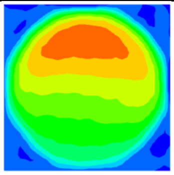	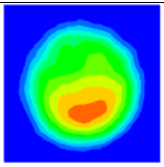	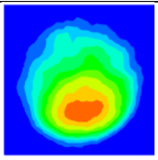	U: 0.093<br>H: 0.087
	27	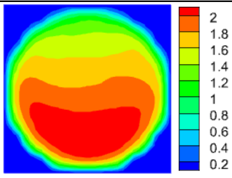	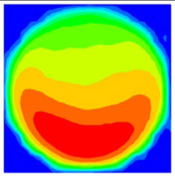	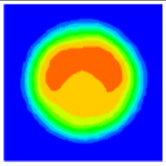	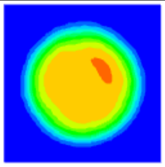	U: 0.086<br>H: 0.083

Mother vessel #	Daughter vessel #s	DSR velocity (mm/s)	ML velocity	DSR hematocrit	ML hematocrit	MAE
34	36	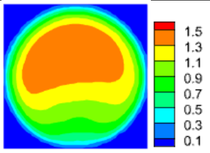	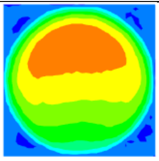	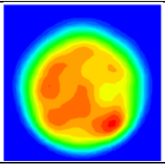	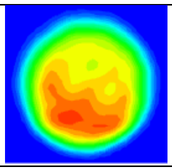	u: 0.12<br>H: 0.13
	37	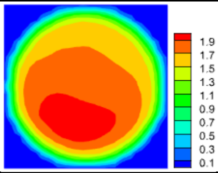	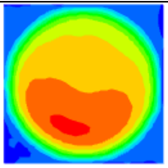	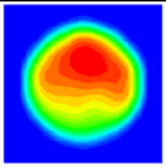	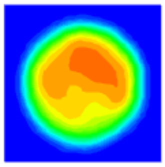	U: 0.12<br>H: 0.088
36	40	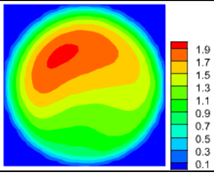	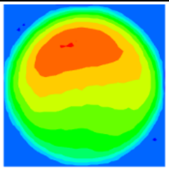	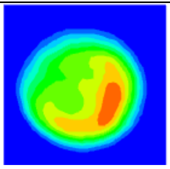	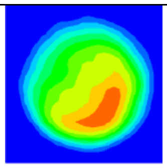	U: 0.115<br>H: 0.082
	41	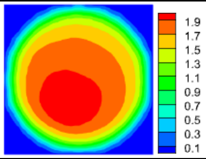	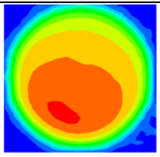	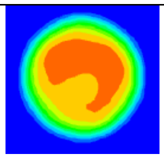	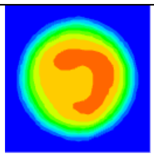	U: 0.1<br>H: 0.057
42	44	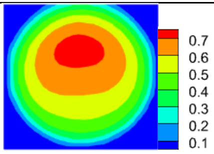	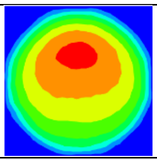	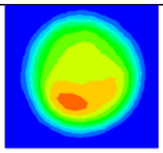	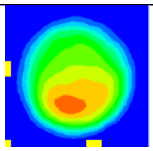	U: 0.072<br>H: 0.06
	45	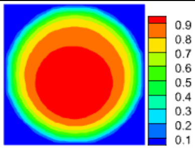	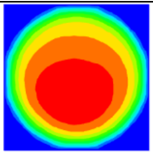	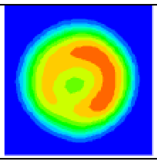	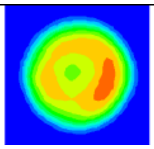	U: 0.1<br>H: 0.068

Fig. S7. 3D prediction of velocity and hematocrit for several individual bifurcations in vasculature B. The last column shows MAE for u and H. For each case, velocity scale is indicated. Hematocrit color ranges from 0 to 1.

Vessel #	DSR velocity	ML velocity			MAE
4	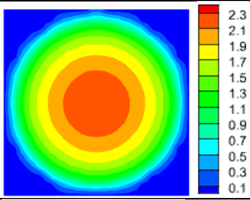	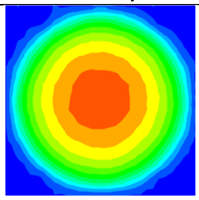	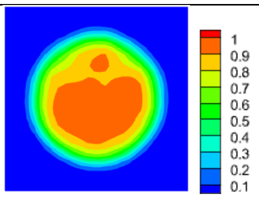	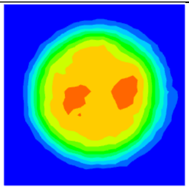	U: 0.07<br>H: 0.13
17	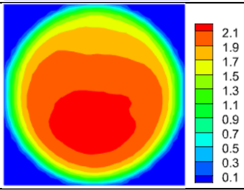	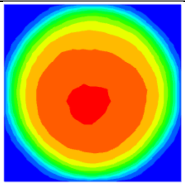	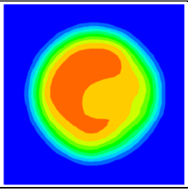	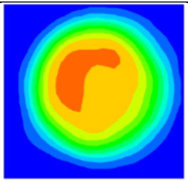	U: 0.12<br>H: 0.065
18	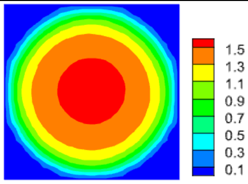	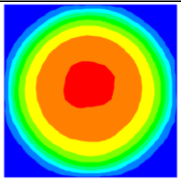	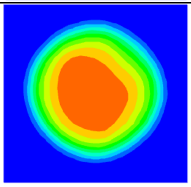	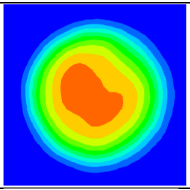	U: 0.069<br>H: 0.06
19	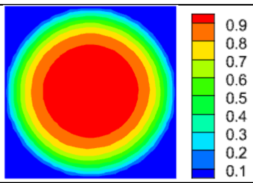	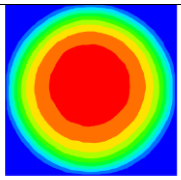	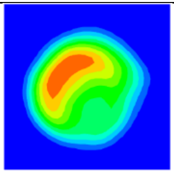	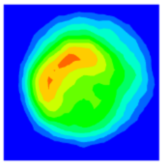	U: 0.064<br>H: 0.072
40	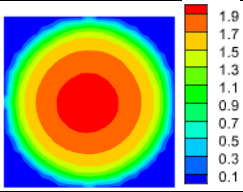	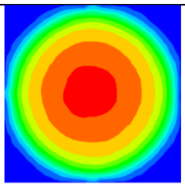	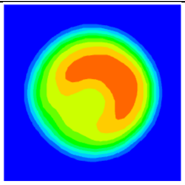	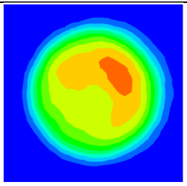	U: 0.075<br>H: 0.045
47	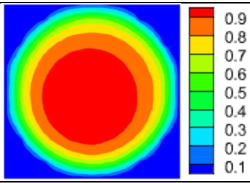	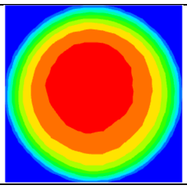	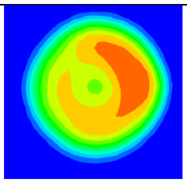	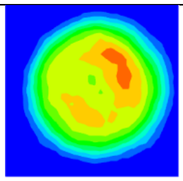	U: 0.098<br>H: 0.11

Fig. S8. Additional 3D predictions for individual vessels. Velocities are in mm/s. For all H plots, contours range from 1 to 0 with 0.1 increments.

Parent vessels	Merged vessel	DSR velocity (mm/s)	ML velocity	DSR hematocrit	ML hematocrit	MAE
12,49	50	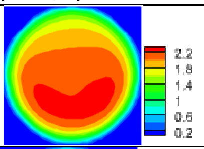	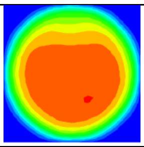	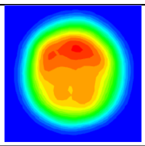	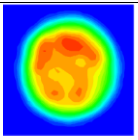	u: 0.11<br>H: 0.08
13,26	51	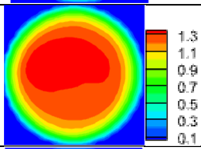	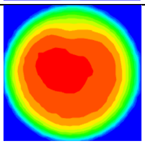	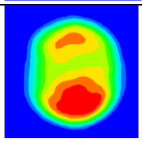	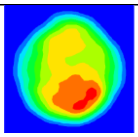	U: 0.12<br>H: 0.06
20,48	52	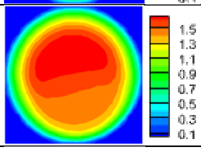	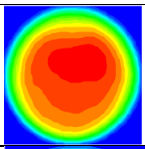	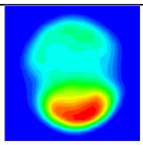	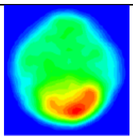	U: 0.13<br>H: 0.085
33,41	55	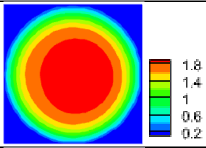	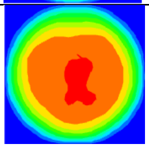	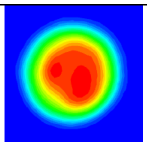	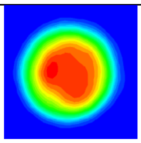	U: 0.14<br>H: 0.064
37,38	56	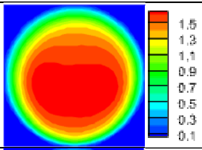	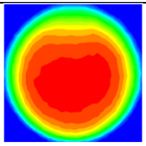	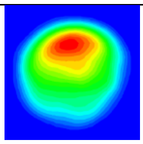	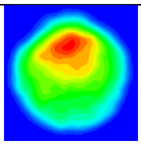	U: 0.1<br>H: 0.072
39,47	57	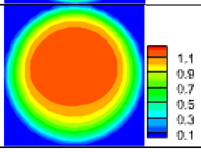	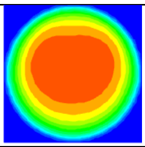	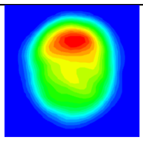	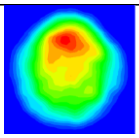	U: 0.1<br>H: 0.077

Fig. S9. Additional 3D predictions for individual mergers. Other details are same as Fig. S8.

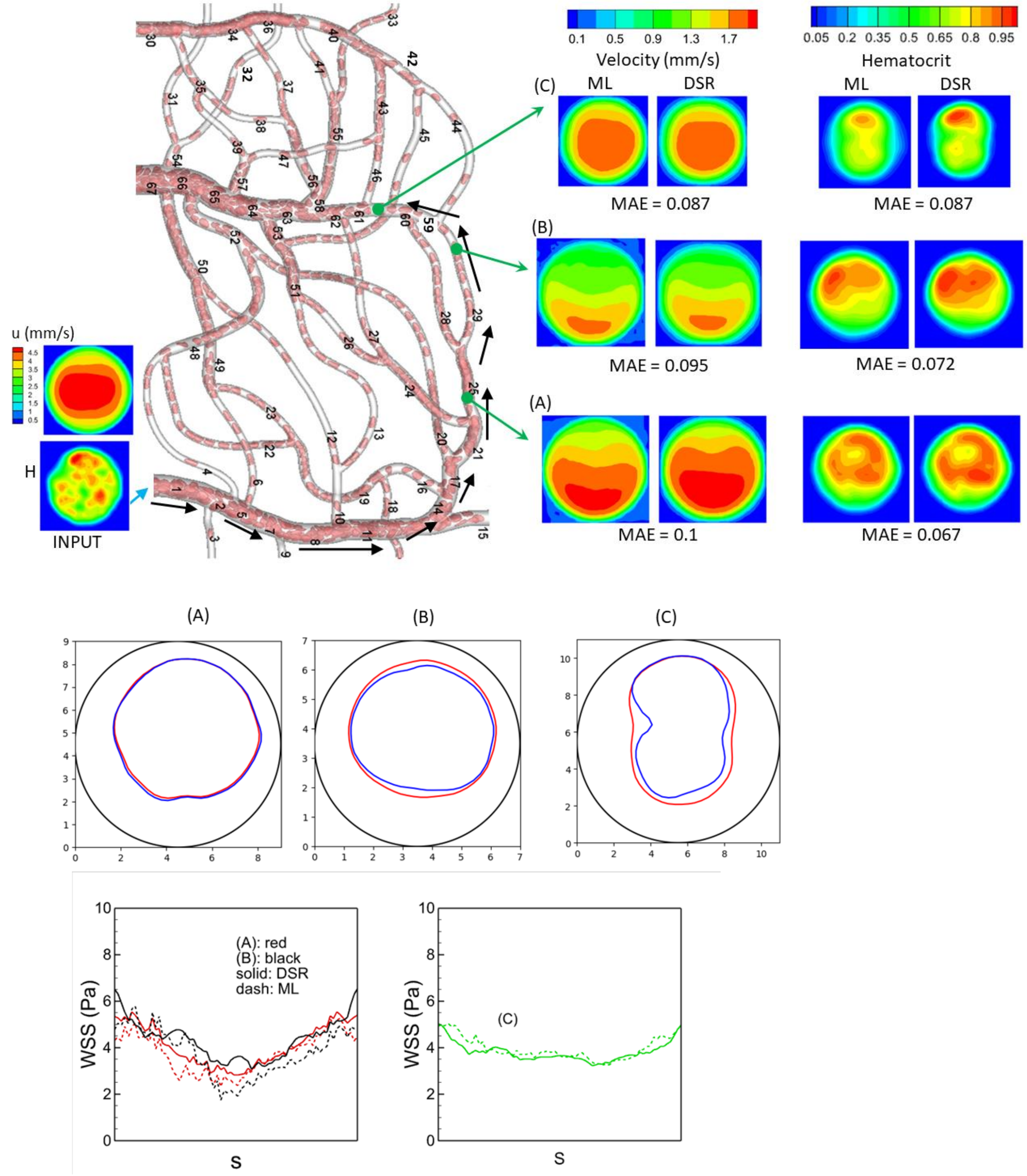

Figure S10. Additional data on vasculature-wide 3D prediction. Black arrows indicate the path. ML and DSR are compared at three locations (A,B,C). Also shown are CFL (red: ML, blue: DSR), and WSS at those locations.

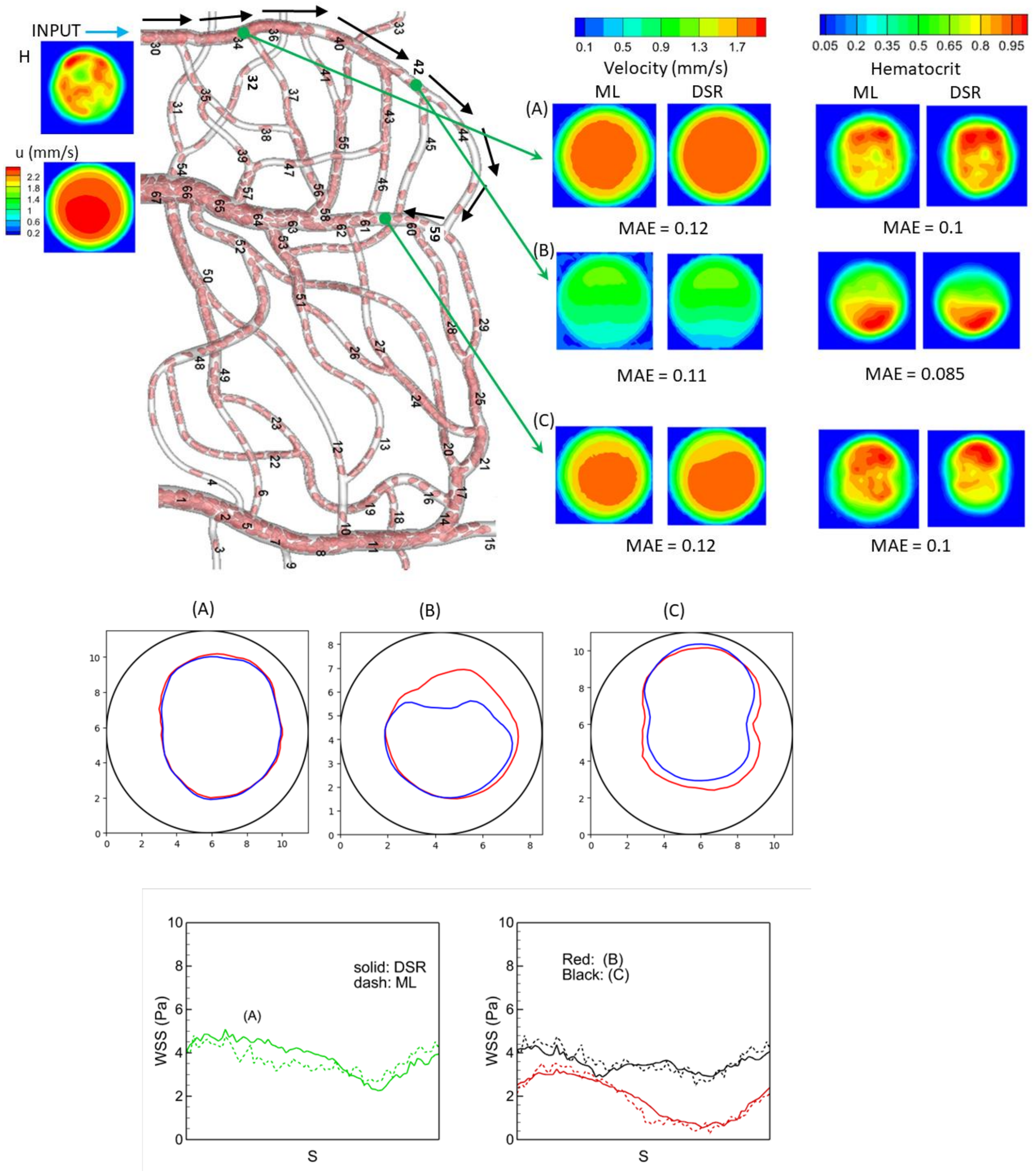

Figure S11. Similar to Fig. S10 but for a different path.

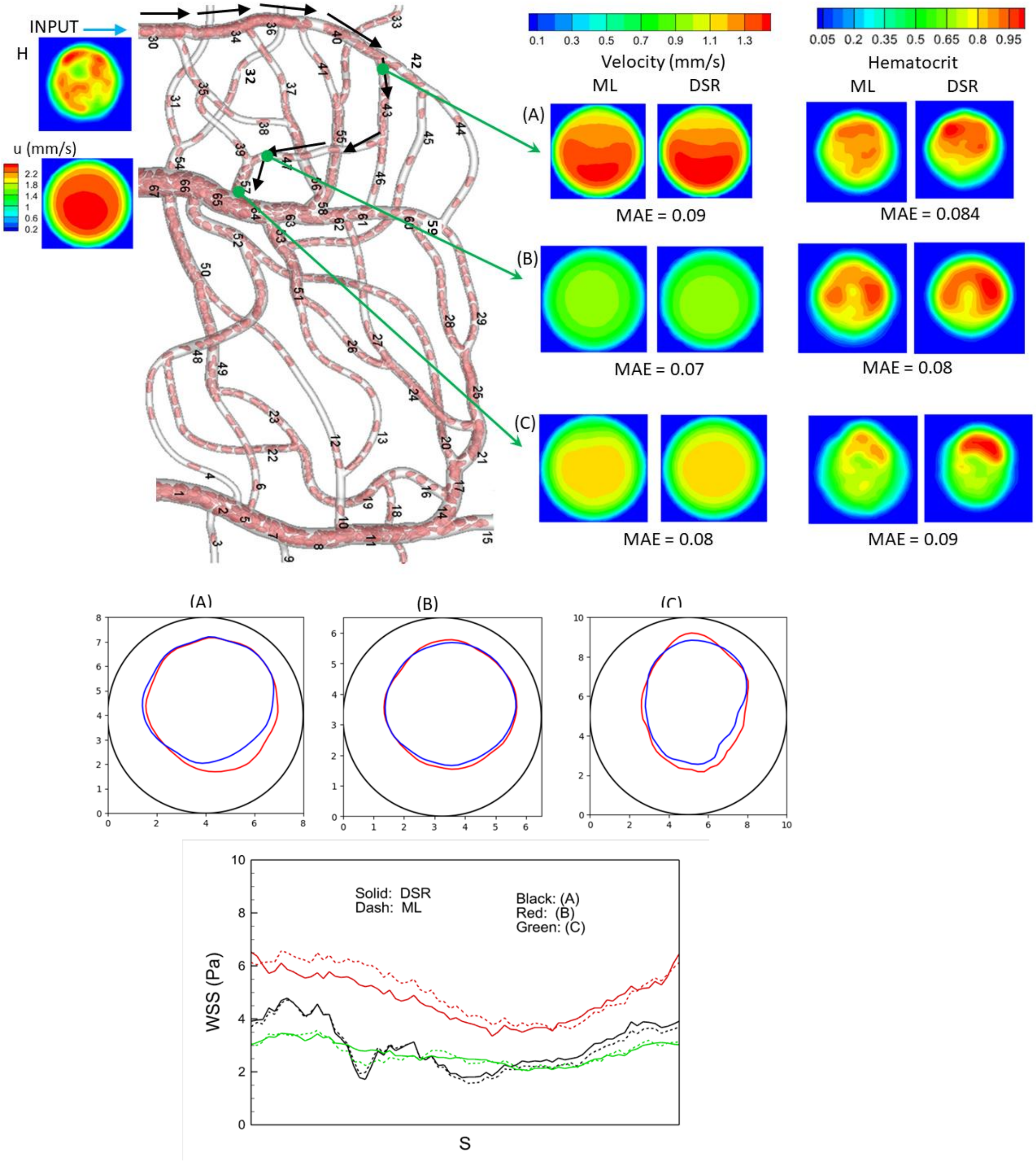

Figure S12. Similar to Figures S10 and S11 but for a different path.

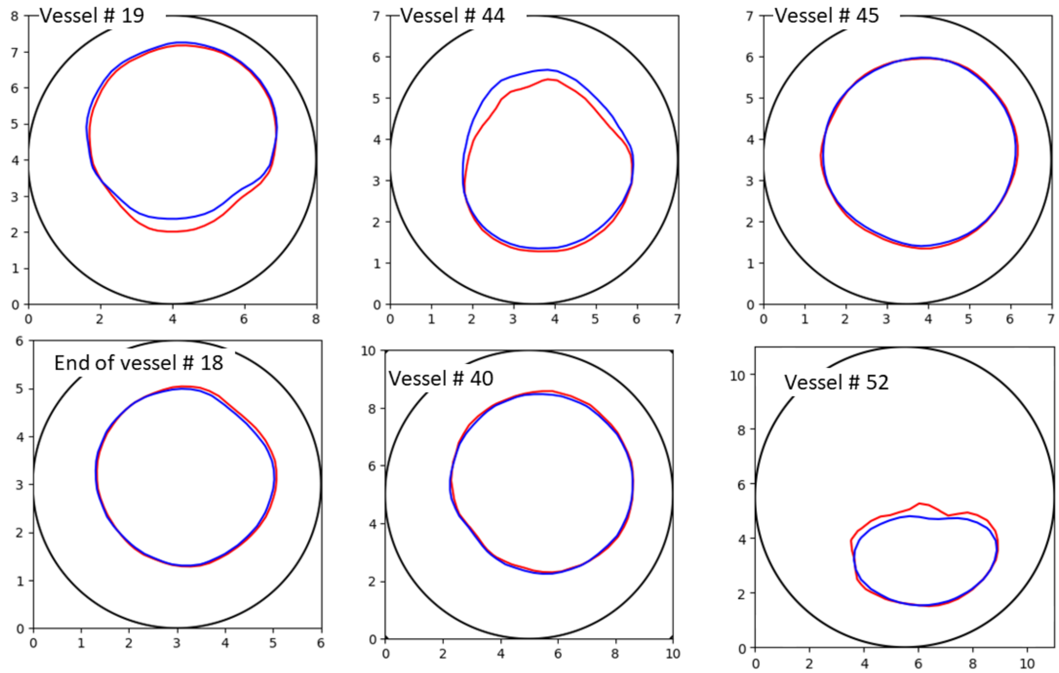

Figure S13. Additional data on CFL from 3D predictions for isolated vascular components. Black: vessel boundary. RBC core is shown in blue for the DSR data and red for the ML prediction.

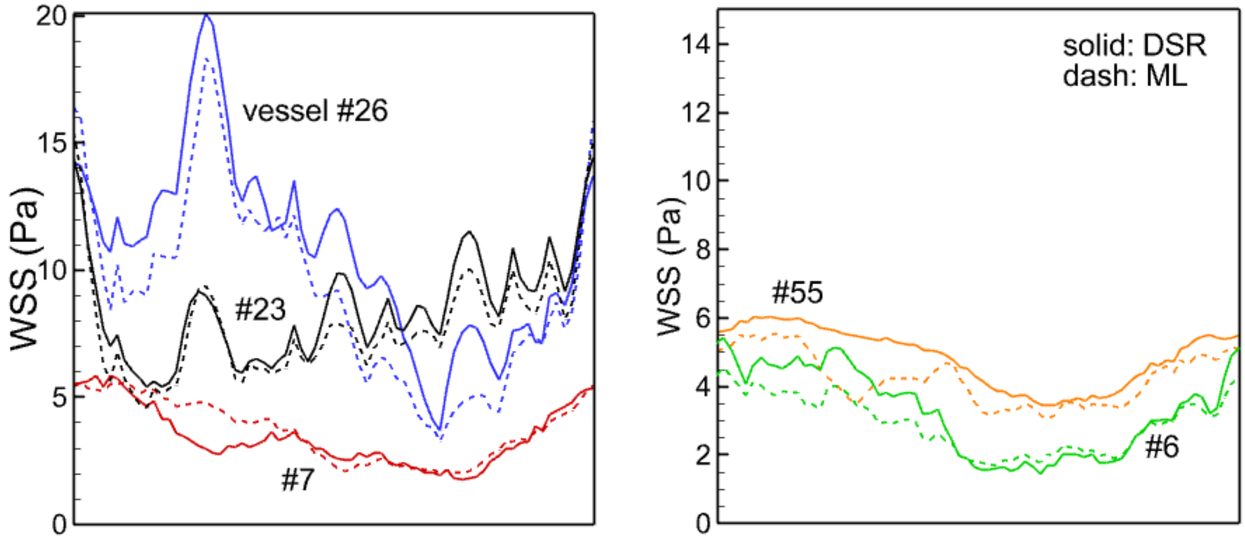

Figure S14. Additional data of WSS prediction in 3D shown for several isolated vessels. Solid line is DSR, dash is ML. horizontal axis indicates the circumference of the vessel at selected locations.
